# Causes of Spontaneous Ignition

**Published:** 1873-09

**Authors:** 


### CAUSES OF SPONTANEOUS IGNITION.

In some letters to the *London Times*, Dr. Attfield points out that animal and vegetable oils differ from mineral oils in relation to combustion, by not catching fire at low temperatures, and in not affording vapor that gives, with certain proportions of air, a mixture which explodes on the approach of flame. But they possess one property of danger from which mineral oils are free, namely, that of being liable to spontaneous ignition, when freely exposed to the air on the surface of a quantity of wool, cloth, paper, cotton, jute, sawdust, lamp-black, ivory-black, ground charcoal, or any similar highly porous substance.

The process of drying which these oils undergo, is not similar to the drying of a wet cloth, where the water simply evaporates, nor to the drying of varnish, in which case it is the spirit that gradually passes into vapor. The drying of oils, on the contrary, is more analogous to the process of combustion, as it is an absorption of oxygen from the atmosphere, the result being the formation of a solid resin. This absorption of oxygen is accompanied with an elevation of temperature, in some cases very considerable. "As a result of direct experiment," Dr. Atfield writes, "I find that paper, cotton, or wool, slightly impregnated with different kinds of oil, and exposed to the air under similar circumstances, rises in temperature from 25 to 200 degrees, according to the variety of oil, the amount of surface in contact with the air, and the duration of exposure." These experiments undoubtedly support the author's conclusion, that in cases where heaps of oiled fabrics are left to oxidize, spontaneous combustion may, and sometimes does, occur.—*Med. and Surg. Reporter.*

